# Non-tobacco nicotine dependence is associated with increased complications following clavicle open reduction internal fixation

**DOI:** 10.1007/s00402-026-06377-2

**Published:** 2026-06-18

**Authors:** Akin A. Adio, Rahul K. Goyal, Abby Skiena, Mohammad Daher, John G. Horneff, Hafiz F. Kassam, Brian W. Hill, Joseph A. Abboud

**Affiliations:** 1 University of Pennsylvania, Philadelphia, USA; 2 Pennsylvania State University, State College, USA; 3 Thomas Jefferson University, Philadelphia, USA; 4 Hoag Orthopedic Institute, Irvine, USA; 5 Palm Beach Orthopaedic Institute, Palm Beach Gardens, USA; 6 Rothman Institute, Philadelphia, USA

**Keywords:** Clavicle fracture, Nicotine, Nonunion, Implant removal

## Abstract

**Background:**

Tobacco dependence is well established to impair bone healing and increase postoperative complications. However, the independent effects of nicotine, separate from combustion-related factors, remain unclear. This study aimed to evaluate the impact of non-tobacco nicotine dependence (NTND) following open reduction and internal fixation (ORIF) of clavicle fractures, including midshaft and lateral fracture subtypes.

**Methods:**

A retrospective cohort study was performed using a large national database. Patients undergoing ORIF of clavicle fractures were identified using procedural and diagnostic codes. Patients with non-tobacco nicotine dependence (e.g., vaping, patches, or gum) were identified and propensity score matched to (1) patients without nicotine exposure and (2) patients with tobacco nicotine dependence. In a secondary analysis, patients were stratified by fracture type (midshaft and lateral), and outcomes were compared between nicotine exposure and no nicotine exposure within each subgroup. Outcomes were assessed at 90 days and 1 year and included medical complications, number of opioid prescriptions, and implant-related outcomes. Risk ratios (RRs) with 95% confidence intervals (CIs) were calculated, with *p* < 0.05 considered significant.

**Results:**

After matching, 936 non-tobacco nicotine users were identified. Non-tobacco nicotine dependence was associated with increased implant removal at 90 days (RR 2.50) and higher opioid prescriptions (*p* < 0.05). At 1-year, non-tobacco nicotine use was associated with increased implant removal (RR 1.74), infection (RR 1.73), and nonunion (RR 1.78) (*p* < 0.05). In direct comparisons between non-tobacco nicotine and tobacco nicotine users (*n* = 897), there were no significant differences in postoperative complications or implant-related outcomes (all *p* > 0.05). In fracture-specific analyses, nicotine use was associated with increased infection (RR 2.17), nonunion (RR 1.42), and implant removal (RR 1.24) in midshaft fractures (*p* < 0.05), with no significant differences observed in lateral fractures.

**Conclusion:**

Non-tobacco nicotine dependence was associated with increased risks of infection, opioid utilization, implant removal, and nonunion following clavicle fracture fixation. These associations were observed predominantly in midshaft fractures, with no significant differences in lateral fractures. These findings suggest that non-tobacco nicotine dependence may carry a risk profile similar to that of tobacco use.

**Level of evidence:**

III.

**Supplementary Information:**

The online version contains supplementary material available at 10.1007/s00402-026-06377-2.

## Introduction

 Clavicle fractures are a relatively common orthopedic injury, accounting for 2.6-5% of all adult fractures [1]. Clavicle fractures most commonly occur in adolescent and young adult males, with peak incidence between ages 13 and 20 [2]. The rate of surgical fixation has been increasing in recent years, with an estimated 445% increase from 2000 to 2022 [3]. Surgical fixation offers the advantage of faster healing, higher union rates, higher short-term patient satisfaction, and a cosmetic appearance more closely resembling typical anatomy [4]. Due to the increase of operative management in clavicle fractures, it is essential we understand the factors that might predispose patients to complications to help guide clinical decision making.

Tobacco use has been well established to increase fracture nonunion rates, heighten infection risk, and impede wound healing [5]. While, cigarette smoking has been declining in the United States for decades, non-tobacco nicotine dependence has been on the rise, partially due to the public perception that alternative nicotine forms, like vapes, are safer [6]. Additionally, nicotine pouch use among US teenagers and young adults nearly doubled between 2023 and 2024, with approximately 13% reporting active use in 2024 [7]. Despite popular belief that non-tobacco nicotine products are safer than smoking, these products may carry similar risks for orthopedic surgery recovery.

Given the increasing use of non-tobacco nicotine especially among adolescents and young adults, who represent a large portion of clavicle fracture patients, its impact on fixation outcomes remains unclear. This study aims to investigate the effect of non-tobacco nicotine dependence on complication rates following open reduction and internal fixation (ORIF) of clavicle fractures, including midshaft and lateral fracture subtypes. We hypothesized that non-tobacco nicotine dependence would significantly impede wound healing and fracture union and predispose patients to infection.

## Methods

### Study design

This retrospective cohort study aimed to assess the risk of postoperative complications at 90 days and 1 year in patients with non-tobacco nicotine dependence undergoing open reduction and internal fixation (ORIF) of clavicle fractures, using data from the TriNetX database. TriNetX aggregates deidentified records from 170 healthcare organizations, covering more than 180 million patients, and provides real-time access to a wide range of electronic health information, including diagnoses, procedures, medications, and laboratory values. Institutional review board approval was not required because only deidentified aggregated data were accessed. We used International Classification of Diseases, 10th Revision (ICD-10) codes to identify patients with clavicle fractures and corresponding procedural codes for ORIF.

### Cohort selection

Patients who underwent ORIF of clavicle fractures were identified using Current Procedural Terminology codes (CPT-23515). These patients were further categorized into three cohorts based on preoperative exposure: non-tobacco nicotine dependence (NTND), tobacco dependence, and controls without nicotine exposure. The NTND cohort was defined as patients with a diagnosis of nicotine dependence excluding cigarette, chewing tobacco, or other combustible tobacco product use (ICD-10: F17.x excluding F17.21, F17.22, F17.29, and Z72.0), consistent with non-combustible nicotine exposure such as e-cigarettes, patches, or gum. The tobacco dependence cohort was defined as patients with a diagnosis of nicotine dependence related to combustible tobacco products (ICD-10: F17.2x and Z72.0). Control patients were those undergoing ORIF of clavicle fractures with no documented history of nicotine dependence or use (ICD-10: Z87.891, F17). The three cohorts were mutually exclusive. Patients with any combustible tobacco code (ICD-10 F17.2x or Z72.0) were excluded from the NTND cohort, so that no patient could contribute to more than one exposure group; this prevented overlap between the tobacco and non-tobacco cohorts and excluded dual users from the NTND group. In a secondary analysis, patients with any nicotine exposure (tobacco or non-tobacco) were compared to those without nicotine exposure and stratified by fracture location (midshaft vs. lateral). Midshaft (ICD-10 S42.02-) and lateral/distal (ICD-10 S42.03-) clavicle fractures were defined using mutually exclusive ICD-10 codes, and propensity score matching was performed separately within each fracture-location subgroup. A complete list of all ICD-10 and CPT codes used to define exposures, fracture locations, and outcomes is provided in Supplementary Table 1. Patients were required to have a documented encounter within 1 year prior to surgery to establish exposure status.

### Statistics

For all outcomes of interest, risk ratios (RRs) and 95% confidence intervals (CIs) were computed using the TriNetX system, with p values less than 0.05 considered statistically significant. Categorical variables were analyzed using the chi-squared test, whereas continuous variables were evaluated using Student t-tests. Propensity score matching was performed in a 1:1 fashion using nearest-neighbor matching without replacement (caliper of 0.1 pooled standard deviations of the logit of the propensity score). The propensity model included age, sex, race, body mass index, hemoglobin A1c, and the comorbidities reported in Table 1 (hypertension; hyperlipidemia; type 2 diabetes mellitus; chronic lower respiratory disease; chronic ischemic heart disease; acute kidney failure and chronic kidney disease; diseases of the liver; disorders of bone density and structure; and concomitant fracture of the ribs, sternum, or thoracic spine). Covariate balance was assessed using standardized mean differences (SMDs) rather than p values alone, with an SMD < 0.1 considered well balanced; all covariates achieved an SMD < 0.1 after matching.

**Table 1:** Baseline Characteristics of Propensity Score–Matched Cohorts

Characteristics		NTND vs. No Nicotine (*n* = 936)			NTND vs. Tobacco (*n* = 897)		
	Cohort	Patients	% Patients	*P*-Value	Patients	% Patients	*P*-Value
Demographics							
Age at Index	NTND Control	936 (37.8 ± 14) 11,805 (36.8 ± 18.4)	100% 100%	0.1127	897 (38 ± 14) 897 (37.8 ± 13.8)	100% 100%	0.6986
White	NTND Control	774 9,649	82.692% 81.737%	0.4657	741 750	82.609% 83.612%	0.5706
Female	NTND Control	238 3,285	25.427% 27.827%	0.1141	233 228	25.975% 25.418%	0.787
Demographics							
Fracture of rib(s), sternum and thoracic spine	NTND Control	364 371	38.889% 39.637%	0.7404	354 322	39.465% 35.897%	0.119
Hypertension	NTND Control	212 219	22.65% 23.397%	0.7007	206 203	22.965% 22.631%	0.8659
Hyperlipidemia, unspecified	NTND Control	94 84	10.043% 8.974%	0.4307	95 89	10.591% 9.922%	0.6406
Pneumothorax and air leak	NTND Control	112 106	11.966% 11.325%	0.6655	108 99	12.04% 11.037%	0.506
Diseases of liver	NTND Control	41 39	4.38% 4.167%	0.8192	42 39	4.682% 4.348%	0.733
Acute kidney failure and chronic kidney disease	NTND Control	38 28	4.06% 3.137%	0.0764	39 36	4.348% 4.013%	0.7234
Type 2 diabetes mellitus	NTND Control	55 49	5.876% 5.235%	0.5449	54 58	6.02% 6.466%	0.6963
Disorders of bone density and structure	NTND Control	42 50	4.487% 5.342%	0.3924	41 38	4.571% 4.236%	0.7299
Chronic ischemic heart disease	NTND Control	45 32	4.808% 3.419%	0.1303	46 43	5.128% 4.794%	0.7443
Chronic lower respiratory diseases	NTND Control	178 180	18.997% 19.21%	0.906	175 176	19.509% 19.621%	0.9525
Labs							
BMI	NTND Control	730 (25.7 ± 5.45) 742 (26.3 ± 5.49)	77.991% 79.274%	0.0506	730 (25.7 ± 5.45) 742 (26.3 ± 5.49)	77.991% 79.274%	0.0506
Hemoglobin A1c	NTND Control	153 (5.7 ± 0.958) 151 (5.71 ± 1.22)	16.346% 16.132%	0.9373	153 (5.7 ± 0.958) 151 (5.71 ± 1.22)	16.346% 16.132%	0.9373

**Table 2:** 90-Day Outcomes and 1 year Following Clavicle Fixation Stratified by Non-Tobacco Nicotine dependence, Tobacco Dependence, and No Nicotine

Non-Tobacco Nicotine vs. No Nicotine (*n* = 936)					NTND vs. Tobacco (*n* = 897)			
90 days								
Infection	35 (3.74%)	22 (2.35%)	1.59 (0.94–2.69)	0.08	35 (3.90%)	33 (3.68%)	1.06 (0.67–1.69)	0.805
Pneumonia	31 (3.30%)	18 (1.92%)	1.82 (0.98–3.27)	0.0683	28 (3.12%)	21 (2.34%)	1.33 (0.76–2.33)	0.311
Wound Disruption	11 (1.18%)	14 (1.50%)	0.79 (0.36–1.72)	0.546	< 10*	< 10*	-	-
Venous Thromboembolism	20 (2.14%)	17 (1.82%)	1.18 (0.62–2.23)	0.618	20 (2.23%)	16 (1.78%)	1.25 (0.65–2.40)	0.501
Pneumothorax	49 (5.24%)	36 (3.85%)	1.36 (0.89–2.07)	0.149	48 (5.35%)	39 (4.35%)	1.23 (0.82–1.86)	0.323
Acute Posthemorrhagic Anemia	28 (2.98%)	26 (2.77%)	1.08 (0.64–1.80)	0.784	22 (2.48%)	21 (2.36%)	1.05 (0.58–1.89)	0.877
Acute Kidney Injury	< 10*	< 10*	-	-	< 10*	< 10*	-	-
Readmission	136 (14.53%)	111 (11.86%)	1.23 (0.97–1.54)	0.088	136 (15.16%)	108 (12.04%)	1.26 (1.00–1.59)	0.054
Implant Removal	40 (4.27%)	16 (1.71%)	2.5 (1.41–4.43)	**0.001**	39 (4.35%)	31 (3.46%)	1.26 (0.79–2.00)	0.329
# Opioid Prescriptions (Mean ± SD)	4.08 ± 5.49	2.81 ± 4.67	-	**< 0.001**	4.13 ± 5.52	3.36 ± 5.73	-	**0.004**
1 year								
Implant Removal	129 (13.74%)	74 (7.88%)	1.74 (1.33–2.29)	**< 0.001**	124 (13.81%)	82 (9.13%)	1.51 (1.16–1.96)	**0.002**
Reoperation	20 (2.13%)	17 (1.81%)	1.18 (0.62–2.23)	0.618	27 (3.01%)	23 (2.56%)	1.17 (0.67–2.03)	0.566
Infection	57 (6.07%)	33 (3.51%)	1.73 (1.14–2.62)	**0.01**	56 (6.24%)	56 (6.24%)	1.00 (0.70–1.42)	1
Nonunion	80 (8.52%)	45 (4.79%)	1.78 (1.25–2.53)	**0.001**	74 (8.24%)	66 (7.35%)	1.12 (0.82–1.54)	0.481

*TriNetX suppresses outcomes with patient counts < 10 for patient anonymity; SD = Standard Deviation

**Table 3:** 1-Year Outcomes Following ORIF of Midshaft and Lateral Clavicle Fractures Stratified by Nicotine Use

Midshaft Fixation (*n* = 2,410)					Lateral Fixation (*n* = 914)			
Outcome	Nicotine *n* (%)	No Nicotine *n* (%)	Risk Ratio (95% CI)	*p*-value	Nicotine *n* (%)	No Nicotine *n* (%)	Risk Ratio (95% CI)	*p*-value
Implant Removal	219 (9.09%)	177 (7.34%)	1.24 (1.02–1.50)	**0.028**	155 (16.96%)	153 (16.74%)	1.01 (0.83–1.24)	0.900
Revision Surgery	68 (2.82%)	67 (2.78%)	1.02 (0.73–1.42)	0.93	31 (3.39%)	31 (3.39%)	1.00 (0.61–1.63)	1.00
Infection	184 (7.64%)	85 (3.53%)	2.17 (1.69–2.78)	**< 0.001**	65 (7.11%)	46 (5.03%)	1.41 (0.98–2.04)	0.063
Nonunion	194 (8.05%)	137 (5.69%)	1.42 (1.15–1.75)	**0.001**	89 (9.74%)	75 (8.21%)	1.19 (0.89–1.59)	0.252

### Outcomes

We evaluated medical complications within 90 days postoperatively, including opioid prescriptions, surgical site infection, venous thromboembolism (deep vein thrombosis and pulmonary embolism), emergency department visits, readmission, pneumothorax, acute posthemorrhagic anemia, and wound disruption. Opioid utilization was defined as the number of opioid prescriptions recorded within 90 days postoperatively. Infection refers to surgical site infection identified by ICD-10 codes for postoperative and surgical-site wound infection. The primary implant-related outcomes assessed at 1 year postoperatively included infection, nonunion, and implant removal. Revision surgery was defined as any subsequent clavicle-related operative procedure, including revision fixation, nonunion surgery, and irrigation and debridement. Fracture-specific analyses were performed to compare outcomes between midshaft and lateral clavicle fractures (Tables 2 and 3). 

## Results

After propensity score matching, 936 patients per cohort were included in the comparison of non-tobacco nicotine dependence and controls, and 897 patients per cohort in the comparison of non-tobacco nicotine dependence and tobacco nicotine dependence. Baseline characteristics were well balanced between groups, with all standardized mean differences < 0.1.

### Non-tobacco nicotine dependence vs. control

Within 90 days, non-tobacco nicotine dependence was associated with higher rates of implant removal (4.27% vs. 1.71%; RR 2.50, *p* = 0.001; absolute risk difference [ARD] 2.6%) and increased opioid prescriptions (4.08 ± 5.49 vs. 2.81 ± 4.67 prescriptions, *p* < 0.001; ARD 1.3 prescriptions). There were no differences in infection (*p* = 0.080), venous thromboembolism (*p* = 0.618), pneumothorax (*p* = 0.149), acute posthemorrhagic anemia (*p* = 0.883), readmission (*p* = 0.091), or wound disruption (*p* = 0.546).

At 1 year, non-tobacco nicotine dependence was associated with higher rates of implant removal (13.74% vs. 7.88%; RR 1.74, *p* < 0.001; ARD 5.9%), infection (6.07% vs. 3.51%; RR 1.73, *p* = 0.010; ARD 2.6%), and nonunion (8.52% vs. 4.79%; RR 1.78, *p* = 0.001; ARD 3.7%). There were no differences in revision surgery (*p* = 0.618).

### Non-tobacco nicotine dependence vs. tobacco nicotine dependence

Within 90 days, there were no significant differences in implant removal (4.35% vs. 3.46%; RR 1.26, *p* = 0.329), infection (3.90% vs. 3.68%; RR 1.06, *p* = 0.805), venous thromboembolism (*p* = 0.501), pneumothorax (*p* = 0.323), pneumonia (*p* = 0.311), or readmission (*p* = 0.054). Non-tobacco nicotine users demonstrated higher mean opioid prescriptions compared with tobacco nicotine users (4.13 ± 5.52 vs. 3.36 ± 5.73, *p* = 0.004).

At 1 year, there were no significant differences in implant removal (*p* = 0.329), infection (*p* = 0.805), nonunion (*p* = 0.431), or revision surgery (*p* = 0.930). Overall, no differences were observed in postoperative complications between groups (all *p* > 0.05).

### Midshaft vs. lateral fractures in nicotine users

At 1 year, nicotine use was associated with higher rates of infection (7.64% vs. 3.53%; RR 2.17, *p* < 0.001; ARD 4.1%), nonunion (8.05% vs. 5.69%; RR 1.42, *p* = 0.001; ARD 2.4%), and implant removal (9.09% vs. 7.34%; RR 1.24, *p* = 0.028; ARD 1.8%) in midshaft fractures. There were no significant differences observed in lateral fractures (implant removal *p* = 0.900, revision *p* = 1.000, infection *p* = 0.063, nonunion *p* = 0.252); the smaller lateral subgroup limits statistical power, so these null findings may reflect inadequate power rather than a true absence of association.

## Discussion

This propensity-matched, retrospective cohort study using the TriNetX database evaluated the association of NTND with 90-day and 1-year postoperative outcomes following ORIF of clavicle fractures. NTND was associated with implant removal and opioid prescriptions at 90 days, as well as increased infection, implant removal, and nonunion at 1 year. When directly comparing NTND with tobacco nicotine users, there were no significant differences in postoperative complications. However, NTND was associated with higher mean opioid utilization. In fracture-specific analyses, increased rates of implant removal, infection, and nonunion were observed in midshaft fractures, with no significant differences seen in lateral fractures.

Infection rates were significantly elevated at 1 year for NTND, with a near-significant increase at 90 days (*p* = 0.08). Lawand et al. similarly found significantly higher rates of skin infections and wound disruption at 90 days following distal radius ORIF [8]. Additonally, Rodgers et al. found significantly elevated infection rates in NTND patients at 90 days following humeral shaft ORIF [9]. Non-tobacco nicotine products may increase infection risk through nicotine-induced proinflammatory cytokines and systemic immune dysregulation, as e-cigarette vapor has been found to reduce neutrophil chemotaxis and lower phagocytosis of bacterial bioparticles [10, 11]. Interestingly, the near significant increase in pneumonia observed in non-tobacco nicotine users (*p* = 0.0683), may also reflect the more direct, perioperative immune-suppressive mechanism stemming from non-tobacco nicotine products. Because the present study did not directly measure nicotine levels, inflammatory markers, or immune function, these mechanisms are offered as possible biological explanations rather than findings demonstrated by the current data. These data suggest that NTND patients undergoing ORIF of clavicle fractures carry a postoperative infection risk similar to that of tobacco users. Therefore, they should be counseled to cease nicotine use prior to surgery and monitored closely for infections in the postoperative period.

At 1 year, there were significantly increased nonunion and implant removal rates observed in the NTND group compared to no nicotine group and significantly increased risk for implant removal compared to tobacco users. This is consistent with Rodgers et al., who similarly found NTND patients had higher odds of nonunion and hardware failure following humeral shaft ORIF [9]. The following bone-biology mechanisms are proposed as plausible explanations rather than direct findings of this study, which did not measure bone biology or osteoblast function. Nicotine inhibits osteoblast proliferation and activates cholinergic anti-inflammatory pathways that suppress tumor necrosis factor-alpha secretion [12, 13]. The greater relative risk of nonunion in NTND users compared to tobacco users may be further attributed to e-cigarette aerosol extracts, which have been shown to impair osteogenic differentiation [14]. NTND products also typically have higher circulating nicotine concentrations due to the nicotine salt formulations commonly used in modern e-cigarettes and pouches [15]. Because implant removal could not be linked to a specific indication in this database, elevated implant-removal rates should be interpreted with caution and may partly reflect elective removal of symptomatic hardware rather than fixation failure. NTND patients may therefore require additional postoperative follow-up and early discussion of revision surgery if nonunion is identified.

Interestingly, when stratifying by fracture location across all nicotine users, significantly increased rates of implant removal, infection, and nonunion at 1 year were only observed in midshaft clavicle fixations, with no significant differences found for lateral fixations. This distinction likely reflects the fundamental differences in the biologic environment of these fracture locations. The clavicle relies on a periosteal blood supply that is vulnerable to compromise with significant displacements, which disproportionately characterizes lateral fractures [16]. Furthermore, there exists several biomechanical challenges of fixation due to the proximity to the acromioclavicular joint and unstable coracoclavicular ligaments [17]. These factors cause lateral fixations to already carry a complication burden high enough to obscure any additional nicotine related cause. In comparison, the midshaft has more robust perfusion and favorable biomechanical conditions, and therefore the vasoconstrictive and anti-osteogenic effects of nicotine become a more dominant source of additional complication risk that can be more easily detected [18]. Clinically, these findings suggest that while nicotine counseling should be given across all clavicle fracture patients, surgical outcomes might be most measurably impacted and modified for midshaft clavicle fixations. In these cases, nicotine cessation could meaningfully reduce what are otherwise expected to be low rates of implant removal, infection, and nonunion. However, this should be interpreted with caution. Because the midshaft and lateral subgroups were propensity matched separately within the platform, formal statistical interaction testing between fracture location and nicotine exposure could not be performed. The apparent difference between subgroups should therefore be regarded as exploratory rather than a confirmed effect-modification finding. The absence of significant associations in lateral fractures may additionally reflect limited statistical power given the smaller lateral subgroup.

Non-tobacco nicotine users required significantly more opioid prescriptions compared to controls. Szilagyi et al. found that smokers required 33.7% more opioids, on average, than nonsmokers within the 24 h postoperative period after abdominal, gynecological, cardiothoracic, orthopedic, and breast or spine surgery [19]. This can be explained by long-term nicotine exposure causing downregulation of opioid receptors. Abrupt perioperative withdrawal may result in hyperalgesia and thus greater opioid consumption [20]. Additionally, the same pathway driving this increased opioid demand also predisposes nicotine users to opioid misuse. Chronic nicotine exposure sensitizes the mesocorticolimbic reward pathway to opioid effects, thus increasing the addictive nature of opioids [21]. The present study found even higher opioid utilization among non-tobacco nicotine users compared with tobacco users. This may reflect differences in nicotine delivery patterns and dependence profiles. Non-combustible nicotine sources can provide more continuous or higher peak nicotine exposure [22]. This can potentially lead to greater receptor desensitization and withdrawal-related hyperalgesia in the perioperative period. Additionally, non-tobacco nicotine users may be less likely to discontinue use prior to surgery due to the perception of these products as safer alternatives, resulting in more pronounced perioperative neuroadaptation and subsequent analgesic demand. Accordingly, non-tobacco nicotine use should be regarded with the same clinical concern as tobacco use.

To our knowledge, this is the first large-scale analysis to evaluate the distinct impacts of tobacco and non-tobacco nicotine dependence on postoperative outcomes following ORIF of clavicle fractures, including midshaft and lateral fracture subtypes. However, there are limitations. Heterogeneity of documentation and coding practices across participating health systems introduces potential for misclassifications of diagnosis, risk factors, and outcomes [23]. Because the NTND cohort was defined using ICD-10 nicotine-dependence codes, this approach cannot reliably distinguish among specific non-combustible products—such as e-cigarettes or vaping, nicotine patches or gum, and nicotine pouches—or identify former smokers using nicotine-replacement therapy. These products differ in nicotine dose and delivery, and grouping them together may obscure clinically meaningful product-specific differences. The use of TriNetX also limits assessment of fracture severity, perioperative medication use, or fixation constructs, among other similarly helpful granular variables that might independently affect postoperative outcomes. In particular, unmeasured confounders most relevant to these outcomes include fracture displacement, comminution, and severity, open versus closed injury, implant type and fixation construct, surgeon and institutional variation, alcohol and substance use disorders, and perioperative medication use including NSAIDs, corticosteroids, and baseline (preoperative) opioid exposure. These factors could not be adjusted for and may affect the observed associations. Furthermore, many factors regarding the nicotine use in the perioperative period, including degree of use, duration of use, and type of non-tobacco nicotine product, are not assessed. Finally, implant removal, although taken as a procedural outcome in our study, may not reflect a true complication, since hardware removal following clavicle ORIF is largely elective due to symptomatic plate irritation in otherwise uncomplicated cases [24]; the database does not record the indication for removal, so symptomatic, elective, and failure-related removals could not be distinguished. Some outcomes were also limited by low event counts, which TriNetX suppresses when fewer than 10 patients are affected, reducing statistical power for less frequent complications.

## Conclusion

Non-tobacco nicotine dependence in patients undergoing ORIF of clavicle fractures was associated with significantly increased rates of infection, nonunion, implant removal, and opioid prescriptions compared with non-nicotine users. Across direct comparisons, NTND demonstrated a risk profile comparable to that of tobacco use for most outcomes. As a retrospective database study, these findings represent associations rather than causal relationships. Our study adds to the growing literature that suggests perioperative non-tobacco nicotine use may confer comparable risks to traditional smoking for postoperative complications following orthopedic fixation. Surgeons should counsel all nicotine users, regardless of delivery method, on elevated risks of postoperative complications prior to clavicle fracture fixation. The growing prevalence of e-cigarettes and nicotine products, especially among younger populations, makes this counseling a particularly important component of preoperative orthopedic care.

## Supplementary Information

Below is the link to the electronic supplementary material.

Supplementary file1

## Data Availability

No datasets were generated or analysed during the current study.
