# Supplementary material for: Non-tobacco nicotine dependence is associated with increased complications following clavicle open reduction internal fixation
Source: Arch Orthop Trauma Surg. 2026 Jun 18;146(1):224. doi: 10.1007/s00402-026-06377-2 (PMC13279569; doi:10.1007/s00402-026-06377-2)
Supplement: Supplementary file 1 — Supplementary file1 [file 402_2026_6377_MOESM1_ESM.docx]

**Supplementary Table 1. ICD-10-CM and CPT Codes Used to Define Exposures, Fracture Locations, and Outcomes**

| **Category** | **Code(s)** | **Description** |
| --- | --- | --- |
| **Procedure (cohort inclusion)** | | |
| ORIF of clavicle fracture | CPT 23515 | Open treatment of clavicular fracture, includes internal fixation, when performed |
| **Exposure cohorts** | | |
| Non-tobacco nicotine dependence (NTND) | ICD-10 F17.x, excluding F17.21, F17.22, F17.29, and Z72.0 | Nicotine dependence consistent with non-combustible use (e.g., e-cigarettes/vaping, patches, gum, pouches); includes F17.20- (nicotine dependence, unspecified / other non-combustible) and excludes all combustible-tobacco subcategories |
| Tobacco (combustible) dependence | ICD-10 F17.2x (F17.21, F17.22, F17.29) and Z72.0 | F17.21 nicotine dependence, cigarettes; F17.22 chewing tobacco; F17.29 other tobacco product; Z72.0 tobacco use |
| Control (no nicotine exposure) | Absence of ICD-10 F17.x and Z72.0; Z87.891 used for exclusion | Patients undergoing clavicle ORIF with no documented nicotine dependence/use; Z87.891 = personal history of nicotine dependence (excluded) |
| **Fracture location (secondary analysis; mutually exclusive)** | | |
| Midshaft clavicle fracture | ICD-10 S42.02- | Fracture of shaft of clavicle (e.g., S42.021–S42.026, with applicable 7th characters) |
| Lateral / distal clavicle fracture | ICD-10 S42.03- | Fracture of lateral end of clavicle (e.g., S42.031–S42.036, with applicable 7th characters) |
| **90-day medical complications** | | |
| Surgical site / postoperative wound infection | ICD-10 T81.4- | Infection following a procedure (e.g., T81.41-, T81.49-) |
| Pneumonia | ICD-10 J12–J18 | Pneumonia, organism unspecified (J18.9) and related categories |
| Wound disruption (dehiscence) | ICD-10 T81.3- | Disruption of wound, not elsewhere classified |
| Venous thromboembolism | ICD-10 I82.4-, I26.- | Deep vein thrombosis (I82.4-) and pulmonary embolism (I26.-) |
| Pneumothorax | ICD-10 J93.-, S27.0- | Pneumothorax, including traumatic |
| Acute posthemorrhagic anemia | ICD-10 D62 | Acute posthemorrhagic anemia |
| Acute kidney injury | ICD-10 N17.- | Acute kidney failure |
| Emergency department visit / readmission | Encounter-based | Inpatient readmission and ED encounters identified by visit type within 90 days |
| Opioid utilization | Medication class (opioid analgesics) | Defined as the number of opioid prescriptions recorded within 90 days postoperatively |
| **Implant-related outcomes (1 year)** | | |
| Implant / hardware removal | CPT 20680; ICD-10 Z47.2 | Removal of implant, deep (CPT 20680); encounter for removal of internal fixation device (Z47.2). Indication not specified in the database. |
| Nonunion | ICD-10 M84.1- | Nonunion of fracture (pseudarthrosis) |
| Implant-related infection | ICD-10 T84.6- | Infection and inflammatory reaction due to internal fixation device of bone |
| Revision surgery / reoperation | CPT 23485, 23515, 10180, 11010–11012 | Any subsequent clavicle-related operative procedure, including revision fixation (23515), repair of nonunion/malunion of clavicle (23485), and irrigation and debridement (10180, 11010–11012) |
